# Impedance-Controlled Molecular Transport Across Multilayer Skin Membranes

**DOI:** 10.3390/membranes16030085

**Published:** 2026-02-27

**Authors:** Slobodanka Galovic, Milena Cukic Radenkovic, Edin Suljovrujic

**Affiliations:** 1 Vinca Institute of Nuclear Sciences-National Institute of the Republic of Serbia, University of Belgrade, Mike Petrovica Alasa 12-14, 522, 11001 Belgrade, Serbia; edin@vin.bg.ac.rs; 2 Institute of Computational Life Sciences, School of Life Sciences and Facility Management, ZHAW Zurich University of Applied Sciences, 8820 Wädenswil, Switzerland; milena.cukic@gmail.com

**Keywords:** skin membrane, transdermal drug delivery, multilayer diffusion, diffusion impedance, cumulative drug uptake

## Abstract

Analytical models of transdermal drug delivery (TDD) often represent deeper skin layers using ideal sink assumptions or phenomenological interfacial resistances. While mathematically convenient, these approaches obscure the physical role of the dermis and hypodermis in controlling molecular transport. Here, we develop an impedance-based analytical model for diffusion across multilayer skin membranes, in which the epidermal barrier is dynamically coupled to a finite diffusive backing layer representing the dermis–hypodermis composite. Diffusion impedance links transport conductivity, storage capacity, and layer thickness, while preserving continuity of concentration and flux at all interfaces. Closed-form expressions in the Laplace domain describe concentration fields and interfacial fluxes, and cumulative drug uptake is computed in the time domain via inverse Laplace transformation. The model identifies distinct short- and long-time transport regimes. Commonly used Dirichlet and Robin boundary conditions emerge as limiting cases but cannot reproduce the regime-dependent behavior of a backing layer. In particular, Robin formulations reduce the backing layer to a constant effective resistance, neglecting its storage capacity and time-dependent impedance. By replacing ad hoc boundary conditions with a physically grounded impedance framework, this approach provides a unified and extensible method for analyzing multilayer transport systems, including extensions to anomalous or memory-dependent diffusion.

## 1. Introduction

**Figure 1:**
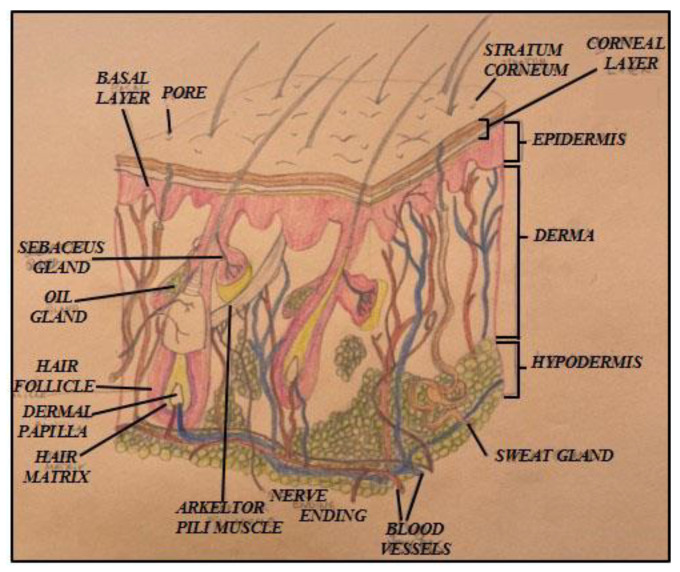
Schematic of skin structure. The stratum corneum (SC) and viable epidermis (VE) form a superficial semipermeable layer acting as the primary molecular barrier. Beneath them, the dermis and hypodermis are thicker, highly vascularized regions that enable systemic distribution of absorbed molecules via the capillary network.

Transdermal drug delivery (TDD) is a noninvasive strategy for local and systemic drug administration, providing controlled dosing, avoidance of first-pass metabolism, and improved patient compliance [1,2,3,4,5]. The skin functions as a multilayer membrane in which molecular diffusion is governed by the coupled properties of its layers rather than by the epidermal barrier alone [6,7,8,9,10] (Figure 1). While the stratum corneum (SC) and viable epidermis (VE) constitute the primary barriers, drug retention and delayed clearance within the dermis and hypodermis significantly influence overall transport kinetics [11,12,13,14,15].

Most analytical models represent deeper skin layers using simplified boundary conditions [16,17]. Commonly, a Dirichlet boundary condition is imposed at the viable epidermis–dermis interface, treating the dermis as an ideal sink where drug molecules are instantly removed by the capillary network [1,12,18,19]. Alternative approaches introduce interfacial resistances or Robin-type boundary conditions to represent imperfect epidermis–dermis coupling [9,10,12,13]. While partially relaxing the ideal sink assumption, these formulations rely on phenomenological parameters and do not explicitly incorporate finite backing-layer storage and geometry. In this paper, we introduce the concept of diffusion impedance to capture the influence of finite diffusivity, storage [20], and backing-layer geometry. Diffusion impedance plays a role analogous to electrical impedance, relating concentration to flux and providing a physically grounded description of interlayer transport [21].

Although the skin exhibits pronounced microstructural heterogeneity [14,17,18], particularly within the stratum corneum, the present analysis focuses on the macroscopic layered structure to isolate backing-layer effects on cumulative uptake. This approximation may be less accurate for highly lipophilic or strongly binding compounds, but it enables analytical tractability and can be extended to incorporate microstructural effects within the impedance framework [22,23,24,25,26,27].

In the present study, the multilayer skin system is represented by a simplified two-layer model: the stratum corneum and viable epidermis form a thin effective membrane, while the dermis and hypodermis are combined into a finite diffusive backing layer. The drug patch serves as an external source, while the distal boundary represents the systemic receiver and may be modeled using absorbing or reflecting conditions.

The proposed impedance-based framework yields three main contributions:

1. It establishes a unified analytical formulation in which backing-layer effects emerge naturally from intrinsic diffusivity, storage capacity, and geometry, rather than from imposed boundary approximations.
2. It demonstrates regime-dependent transport behavior: at short times the backing layer behaves as diffusively thick and screens the distal boundary, whereas at long times it becomes diffusively thin and cumulative uptake is governed by the distal boundary condition.
3. It shows that Dirichlet and Robin boundary conditions arise as limiting cases of simplified impedance and therefore cannot reproduce the full temporal dynamics of a finite diffusive layer.

This perspective shifts the interpretation of permeability from a fixed material constant to an emergent, impedance-controlled, time-dependent property of the coupled system. The practical implications of these dynamic backing-layer effects—guiding ex vivo experiments, controlled-release patch design, and microfluidic measurements—are discussed.

Although TDD serves as a physiologically relevant example, the analytical structure applies generally to multilayer systems where a thin barrier is dynamically coupled to a finite diffusive layer [28,29,30,31].

The remainder of this paper is organized as follows. Section 2 presents the theoretical formulation and introduces diffusion impedance and transfer-matrix analysis. Section 3 derives analytical expressions for cumulative drug uptake and discusses the relevant transport regimes. The main conclusions are summarized in Section 4 and Section 5.

## 2. Theory: The Concept of Diffusion Impedance

### 2.1. Model Description

We base our analysis on Fickian diffusion and focus on the macroscopic layered structure of the skin, intentionally neglecting the heterogeneous and fractal microstructure of individual layers. This deliberate simplification allows us to isolate and analyze the role of macroscopic (layered) heterogeneity independently of microstructural effects, which may give rise to anomalous, subdiffusive, or memory-dependent transport regimes [17]. By design, this means that our model is not intended to capture the fine-scale microstructural pathways or binding heterogeneities that could dominate transport for certain compounds.

To quantify the influence of finite dermal conductivity and storage capacity on transdermal transport, we consider a two-layer analytical model. The stratum corneum (SC) and viable epidermis (VE) are treated as a thin effective barrier, while the dermis and hypodermis are represented as a finite backing layer.

**Figure 2:**
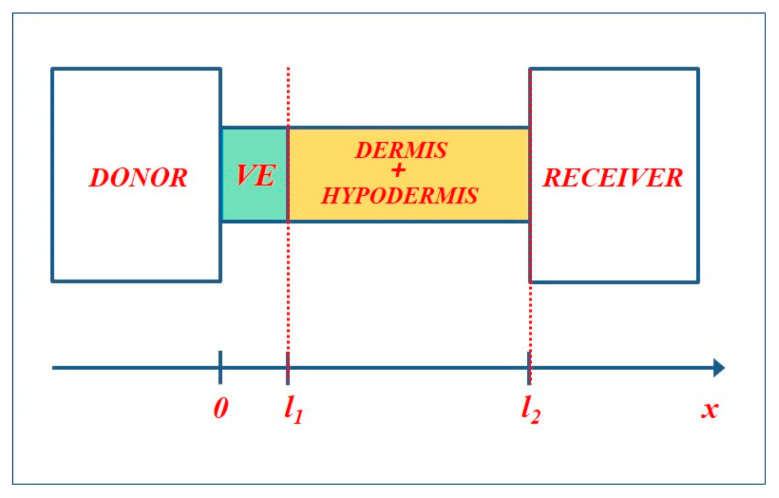
The geometry of the problem.

The transdermal patch acts as an external source at the membrane surface, while the distal boundary of the backing layer corresponds to the interface with the systemic receiver, which can be modeled as either an ideal sink or a perfectly reflecting boundary (see Figure 2). Continuity of both concentration and diffusive flux is imposed at the interface between the barrier and the backing layer, ensuring physically consistent coupling. Within this formulation, the multilayer system can be viewed as a sequence of coupled transport elements, whose interaction is most naturally described using transfer matrices and impedance concepts [21,27].

The initial condition assumes no drug molecules present in the system prior to patch application, consistent with standard TDD experiments.

The mathematical formulation of the problem is given by the following system of partial differential equations.

For layer 1 (viable epidermis, VE):

(1) $$\frac{\partial^{2}n_{1}(x,t)}{\partial x^{2}}-\frac{\gamma_{1}}{D_{1}}\frac{\partial n_{1}(x,t)}{\partial t}=0$$

(2) $$j_{1}(x,t)=-D_{1}\frac{\partial n(x,t)}{\partial x}$$

For layer 2 (dermis + hypodermis):

(3) $$\frac{\partial^{2}n_{2}(x,t)}{\partial x^{2}}-\frac{\gamma_{2}}{D_{2}}\frac{\partial n_{2}(x,t)}{\partial t}=0$$

(4) $$j_{2}(x,t)=-D_{2}\frac{\partial n_{2}(x,t)}{\partial x}$$

where *γ*_1_ and *γ*_2_ denote the mass storage capacities of the viable epidermis and dermis, respectively, and *D*_1_ and *D*_2_ are the corresponding diffusion coefficients.

The donor phase is described by a constant concentration, which is applied at the moment the transdermal patch is placed on the skin [1]:

(5) $$n_{1}(x=0,t)=n_{0}h(t)$$

At the distal boundary of the backing layer, either an ideal receiver (ideal sink) is assumed,

(6) $$n_{2}(x=l_{1}+l_{2},t)=0$$

or a completely impermeable boundary (adiabatic condition):

(7) $$j_{2}(x=l_{1}+l_{2},t)=0$$

At the heterointerface between the barrier and the backing layer, continuity of concentration and diffusive flux is imposed:

(8) $$n_{1}(x=l_{1},t)=n_{2}(x=l_{1},t)$$

(9) $$j_{1}(x=l_{1},t)=j_{2}(x=l_{1},t)$$

which represents the most physically justified choice for coupling between layers.

It is further assumed that no drug molecules are present in the system prior to patch application, leading to zero initial conditions:

(10) $$n_{1}(x,t=0)=n_{2}(x,t=0)=0$$

### 2.2. Laplace Formulation and Diffusion Impedance

Applying the Laplace transform to the governing equations yields the following system describing the two-layer structure:

(11) $$\frac{d^{2}{\bar{n}}_{k}(x,s)}{dx^{2}}-{\bar{\sigma }}_{k}^{2}\bar{n}(x,s)=0$$

(12) $${\bar{j}}_{k}(x,s)=-\frac{1}{{\bar{\sigma }}_{k}{\bar{Z}}_{ck}}\frac{d{\bar{n}}_{k}(x,s)}{dx}$$

where

(13) $${\bar{\sigma }}_{k}=\sqrt{\frac{\gamma_{k}}{D_{k}}}\sqrt{s}$$

(14) $${\bar{Z}}_{ck}=\frac{1}{\sqrt{\gamma_{k}D_{k}}}\frac{1}{\sqrt{s}}$$

with *k* = 1, 2.

In this representation, each layer is characterized by an effective propagation coefficient and a characteristic diffusion impedance, allowing the multilayer structure to be treated analogously to a cascaded transport network [27].

The boundary condition at the donor interface corresponds to a constant drug concentration applied by the patch:

(15) $${\bar{n}}_{1}(x=0,s)=n_{0}\frac{1}{s}$$

At the distal side of the backing layer, either an ideal sink (Dirichlet) or a reflecting (Neumann) boundary condition can be used,

(16) $${\bar{n}}_{2}(x=l_{1}+l_{2},s)=0$$

(17) $${\bar{j}}_{2}(x=l_{1}+l_{2},s)=0$$

while continuity conditions are enforced at the membrane–backing interface:

(18) $${\bar{n}}_{1}(x={l_{1}}^{-},s)={\bar{n}}_{2}(x={l_{1}}^{+},s)$$

(19) $${\bar{j}}_{1}(x={l_{1}}^{-},s)={\bar{j}}_{2}(x={l_{1}}^{+},s)$$

Solving this system provides analytical expressions for concentration and flux at the interface.

For *x* < *l*_1_ we obtained

(20) $$\left[\begin{array}{c} {\bar{n}}_{1}(x,s)\\ {\bar{j}}_{1}(x,s) \end{array}\right]=\left[\begin{array}{cc} ch({\bar{\sigma }}_{1}x) & -{\bar{Z}}_{c1}sh({\bar{\sigma }}_{1}x)\\ -\frac{1}{{\bar{Z}}_{c1}}sh({\bar{\sigma }}_{1}x) & ch({\bar{\sigma }}_{1}x) \end{array}\right]\left[\begin{array}{c} n_{0}\\ \bar{j}(0,s) \end{array}\right]$$

For *x* > *l*_1_

(21) $$\left[\begin{array}{c} {\bar{n}}_{2}(x,s)\\ {\bar{j}}_{2}(x,s) \end{array}\right]=\left[\begin{array}{cc} ch({\bar{\sigma }}_{2}(x-l_{1})) & -{\bar{Z}}_{c2}sh({\bar{\sigma }}_{2}(x-l_{1}))\\ -\frac{1}{{\bar{Z}}_{c2}}sh({\bar{\sigma }}_{2}(x-l_{1})) & ch({\bar{\sigma }}_{2}(x-l_{1})) \end{array}\right]\left[\begin{array}{c} {\bar{n}}_{1}(l_{1},s)\\ {\bar{j}}_{1}(l_{1},s) \end{array}\right]$$

To explicitly account for the effect of the backing, we define the diffusion impedance of the backing layer as

(22) $${\bar{Z}}_{2}=\frac{{\bar{n}}_{1}\left(l_{1},s\right)}{{\bar{j}}_{1}(l_{1},s)}$$

where $\bar{n}\left(l_{1},s\right)$ and $\bar{j}(l_{1},s)$ are the Laplace domain concentration and flux at the interface between the membrane and the backing, respectively.

This definition formally encapsulates the entire response of the backing layer—including its material properties, thickness, and distal boundary condition—into a single impedance function.

Substituting Equation (22) into Equation (21) and solving the resulting transfer-matrix equation yields, with boundary conditions, Equations (15)–(17), the total flux and concentration at the barrier–backing interface:

(23) $$\bar{j}(l_{1},s)=n_{0}\frac{1}{s}\frac{1}{{\bar{Z}}_{c1}sh({\bar{\sigma }}_{1}l_{1})+{\bar{Z}}_{2}ch({\bar{\sigma }}_{1}l_{1})}$$

(24) $$\bar{n}(l_{1},s)=n_{0}\frac{1}{s}\frac{{\bar{Z}}_{2}}{{\bar{Z}}_{c1}sh({\bar{\sigma }}_{1}l_{1})+{\bar{Z}}_{2}ch({\bar{\sigma }}_{1}l_{1})}$$

The backing impedance can be obtained by solving the differential equation (Equation (21)) with boundary condition at the distal side of the deeper layer of skin, given by Equation (16) for the reflection or by Equation (17) for the antireflection condition.

By applying Equation (21) and the Dirichlet boundary condition (reflective) at the distal side of the backing layer (Equation (16)), we obtain the following expression for the diffusion impedance of the backing:

(25) $${\bar{Z}}_{2}=\frac{{\bar{n}}_{1}(l_{1},s)}{{\bar{j}}_{1}(l_{1},s)}={\bar{Z}}_{c2}\frac{sh({\bar{\sigma }}_{2}l_{2})}{ch({\bar{\sigma }}_{2}l_{2})}$$

Alternatively, by applying Equation (21) together with the Neumann boundary condition (antireflective) (Equation (17)), the diffusion impedance becomes

(26) $${\bar{Z}}_{2}=\frac{{\bar{n}}_{1}(l_{1},s)}{{\bar{j}}_{1}(l_{1},s)}={\bar{Z}}_{c2}\frac{ch({\bar{\sigma }}_{2}l_{2})}{sh({\bar{\sigma }}_{2}l_{2})}$$

It is important to note that both the characteristic diffusion impedance and the effective propagation coefficient depend on the transport properties of the backing layer, such as conductivity and mass storage capacity (Equations (13) and (14)). In cases where the relationship between thermodynamic scalar fields and their fluxes deviates from classical Fickian behavior—such as in subdiffusive, fractional, or wave-like transport processes [22,23,24,25,26]—these quantities acquire modified frequency dependence determined by the fractional order of differentiation or relaxation times associated with inertial memory effects.

This observation highlights the generality of the proposed framework: by appropriately redefining the coefficient of propagation (Equation (13)) and characteristic impedance (Equation (14)), the same analytical structure remains valid for fractional, subdiffusive, or wave-like transport models, without altering the macroscopic transfer-matrix formulation [18,27].

## 3. Cumulative Drug Amount and Effective Transmission

A key quantity in transdermal drug delivery (TDD) applications is the cumulative amount of drug passing through the epidermal barrier, defined as the time integral of the diffusive flux at the VE–dermis interface [1,12,17]:

(27) $$Q(t)=A\int_{0}^{t}j(x=l_{1},t)$$

where *A* denotes the cross-sectional area of the patch application site. The quantity *Q*(*t*) has the dimension of substance amount and represents the total number of drug molecules that have crossed the epidermal barrier up to time t.

To evaluate this quantity for the two-layer system shown in Figure 2, we first apply the Laplace transform to Equation (27). The spectral representation of the cumulative amount of drug transmitted across the barrier is given by

(28) $$\bar{Q}(s)=\frac{A}{s}{\bar{j}}_{1}(x=l_{1},s)$$

Substituting the interface flux obtained in Equation (23) into Equation (28) yields

(29) $$\bar{Q}(s)=An_{0}\frac{1}{s^{2}}\frac{1}{{\bar{Z}}_{c1}sh({\bar{\sigma }}_{1}l_{1})+{\bar{Z}}_{2}ch({\bar{\sigma }}_{1}l_{1})}$$

As evident from Equation (29), the cumulative amount of drug entering the vascularized layers of the skin depends not only on the properties of the epidermal barrier (thickness, conductivity, and storage capacity) but also on the transport properties of the backing layer, fully encoded exclusively through its diffusion impedance. This formulation ensures that both the barrier and backing-layer contributions to drug uptake are explicitly accounted for, providing a physically grounded basis for subsequent analytical evaluation in the Laplace domain.

The inverse Laplace transform provides the time-dependent cumulative drug amount *Q*(*t*), which naturally captures the influence of layer thicknesses, transport parameters, and distal boundary conditions, without introducing artificial interfacial resistances commonly used in classical TDD models [9,10,13].

### 3.1. Ideal Receiver as a Limiting Case

This limiting case highlights the scenario where distal boundary conditions are fully screened by the backing layer.

If the backing layer is assumed to act as an ideal receiver (D2→∞), the corresponding diffusion impedance vanishes, ${\bar{Z}}_{c2}\to 0$ (Equation (14)), and Equation (29) reduces to

(30) $$\bar{Q}(s)=n_{0}\frac{1}{s^{2}}\frac{1}{{\bar{Z}}_{c1}sh({\bar{\sigma }}_{1}l_{1})}$$

This is equivalent to imposing a Dirichlet boundary condition at the VE–dermis interface [1]. In this regime, *Q*(*t*) initially increases linearly and then gradually saturates, with a characteristic time scale determined by the barrier conductivity, storage capacity, and thickness.

### 3.2. Diffusively Thin and Diffusively Thick Layer Regimes of Backing Layer

The behavior of the backing layer depends on the ratio of layer thickness to characteristic diffusion length. A layer is diffusively thin when $l_{k}<<\mu_{k}$ and diffusively thick when the opposite inequality holds $l_{k}>\mu_{k}$, where the characteristic diffusion length is determined by Equation (13):

(31) μk=1Reσ¯k

Physically, this distinction compares the layer thickness to the characteristic distance over which concentration disturbances can propagate during the observation time.

Considering that

(32) $$\frac{sh(\sqrt{x})}{\sqrt{x}}=1+\frac{x}{3!}+\frac{x^{2}}{5!}+\ldots =1+\frac{x}{6}+\frac{x^{2}}{120}+\ldots$$

(33) $$ch(\sqrt{x})=1+\frac{x}{2!}+\frac{x^{2}}{4!}+\ldots =1+\frac{x}{2}+\frac{x^{2}}{24}+\ldots$$

(34) $$\frac{th(\sqrt{x})}{\sqrt{x}}=\frac{1+\frac{x}{3!}+\frac{x^{2}}{5!}+\ldots }{1+\frac{x}{2!}+\frac{x^{2}}{4!}+\ldots }=\frac{1+\frac{x}{6}+\frac{x^{2}}{120}+\ldots }{1+\frac{x}{2}+\frac{x^{2}}{24}+\ldots }$$

for geometrically thin layers satisfying $l_{k}<<\mu_{k}\sqrt{6}$, the following approximations hold:

(35) $$\frac{sh(\sqrt{x})}{\sqrt{x}}\approx 1$$

(36) $$ch(\sqrt{x})\approx 1+\frac{x}{2}$$

(37) $$\frac{th(\sqrt{x})}{\sqrt{x}}\approx \frac{1}{1+\frac{x}{2}}$$

In contrast, for geometrically thick layers, $l_{k}>>\mu_{k}$,

(38) $$ch(\sqrt{x})\approx sh(\sqrt{x})$$

can be assumed.

These approximations allow for classification of backing-layer behavior, which directly influences the sensitivity of cumulative drug uptake to distal boundary conditions.

### 3.3. Application to Epidermal Barrier and Backing Layer

For typical skin parameters [13,32,33], the viable epidermis (VE) is always diffusively thin over the relevant time scale, while the dermis + hypodermis (backing layer) behaves as: (a) thick at short times (for high harmonics), meaning distal boundaries are irrelevant, and (b) thin at long times (for low harmonics), where distal boundaries influence *Q*(*t*).

In the short-time limit, the backing-layer diffusion impedance reduces to its characteristic value ${\bar{Z}}_{c2}={\bar{Z}}_{2}$ and Equation (29) is reduced to

(39) $$\bar{Q}(s)=An_{0}\frac{1}{s^{2}}\frac{1}{{\bar{Z}}_{c1}sh({\bar{\sigma }}_{1}l_{1})+{\bar{Z}}_{c2}ch({\bar{\sigma }}_{1}l_{1})}$$

Equation (39) applies to a backing layer of finite thickness in the quasi-static regime. At high frequencies (short-time limit), the backing layer becomes diffusively thin and the distal boundary conditions at the skin–systemic interface become explicitly visible through Equations (25) and (26).

This emphasizes that distal boundaries are effectively invisible in this regime, confirming low sensitivity to distal BCs.

### 3.4. Characteristic Transport Regimes

Assuming a geometrically thin epidermal barrier, four characteristic transport regimes can be identified:

**(1)**  **Backing layer as an ideal receiver**

Substituting Equation (35) into Equation (30) and using Equations (13) and (14), the spectral function of the cumulative amount becomes

(40) $$\bar{Q}(s)=n_{0}A\frac{D_{1}\gamma_{1}}{l_{1}}\frac{1}{s^{2}}$$

Applying inverse Laplace transformation on Equation (40) yields [34]

(41) $$Q(t)=n_{0}A\frac{D_{1}\gamma_{1}}{l_{1}}th(t)$$

As seen from Equations (40) and (41), the cumulative drug amount is independent of both the backing-layer thickness and the distal boundary conditions. It depends exclusively on the barrier properties.

**(2)**  **Diffusively thick backing layer (long-time limit)**

Substituting Equations (35)–(38) into Equation (39) and using Equations (13) and (14), the cumulative amount is described by

(42) $$\bar{Q}(s)=n_{0}Aa_{2}\frac{1}{s^{2}}\frac{\sqrt{s}}{s+b_{1}\sqrt{s}+b_{2}}$$

with coefficients defined in

(43) $$a_{2}=\frac{2\gamma_{2}D_{1}\sqrt{D_{2}}}{l_{1}^{2}},\;b_{1}=\frac{2\gamma_{2}\sqrt{D_{2}}}{\gamma_{1}l_{1}},\;\mathrm{and}\;b_{2}=\frac{2D_{1}}{l_{1}^{2}}$$

Due to the presence of irrational poles and zeros, the inverse Laplace transform is evaluated via the substitution $p=\sqrt{s}$ [35,36]

(44) $$\bar{Q}(s)=n_{0}Aa_{2}\frac{1}{p^{3}}\frac{1}{p^{2}+b_{1}p+b_{2}}$$

leading to

(45) $$Q(t)=n_{0}Aa_{2}\left[\frac{K_{1}}{\sqrt{\pi t}}+K_{2}+K_{5}e^{\lambda_{1}^{2}t}erfc\left(\lambda_{1}\sqrt{t}\right)-K_{6}e^{\lambda_{2}^{2}t}erfc\left(\lambda_{2}\sqrt{t}\right)\right]$$

with coefficients given by Equations (44) and (45),

(46) $$K_{5}=\frac{K_{3}\left(-\lambda_{1}\right)+K_{4}}{\lambda_{2}-\lambda_{1}},K_{6}=\frac{K_{3}\left(-\lambda_{2}\right)+K_{4}}{\lambda_{2}-\lambda_{1}}$$

(47) $$K_{1}=-\frac{2b_{1}}{b_{2}},\;K_{2}=\frac{2}{b_{2}},\;K_{3}=\frac{2b_{1}}{b_{2}^{2}},\;K_{4}=\frac{2\left(b_{1}^{2}-b_{2}\right)}{b_{2}^{2}},\;\lambda_{1/2}=\frac{b_{1}\pm \sqrt{b_{1}^{2}-4b_{2}}}{2}$$

In this regime, the cumulative drug amount exhibits a coupled dependence on both the barrier and backing-layer properties, while remaining insensitive to the distal boundary condition.

**(3)**  **Diffusively thin backing layer with ideal receiver (Dirichlet boundary)—short-time limit**

Starting from Equations (25) and (29) and applying the expansions (Equations (35)–(38)), the spectral function becomes

(48) $$\bar{Q}(s)=n_{0}A\frac{D_{1}\gamma_{1}}{l_{1}}\frac{1}{s^{2}}\frac{s+a_{3}}{s+b_{3}}$$

with parameters defined in Equation (49):

(49) $$a_{3}=\frac{D_{2}\gamma_{2}}{l_{1}l_{2}\gamma_{1}}\;\mathrm{and}\;b_{3}=\frac{D_{2}\gamma_{2}l_{1}+D_{1}\gamma_{1}l_{2}}{\gamma_{1}l_{2}l_{1}^{2}}$$

Inverse Laplace transformation performed by the method of partial fraction [37] yields

(50) $$Q(t)=n_{0}A\frac{D_{1}\gamma_{1}}{l_{1}}\left[\frac{a_{3}}{b_{3}}t+\frac{a_{3}-b_{3}}{b_{3}^{2}}\left(e^{-b_{3}t}-1\right)\right]h(t)$$

The slope and characteristic time scale of increase depend on the transport properties of both the barrier and the backing layer.

**(4)**  **Diffusively thin backing layer with insulating distal boundary (Neumann boundary)—short-time limit**

Starting from Equations (26) and (29) and applying Equations (35)–(37), the corresponding spectral function becomes

(51) $$\bar{Q}(s)=n_{0}A\frac{2D_{1}D_{2}}{\gamma_{1}l_{2}l_{1}^{2}}\frac{1}{s}\frac{1}{s^{2}+b_{4}s+b_{5}}$$

with coefficients

(52) $$b_{4}=\frac{l_{1}^{2}\gamma_{1}D_{2}+2l_{1}l_{2}\gamma_{2}D_{2}+l_{2}^{2}\gamma_{2}D_{2}}{l_{1}^{2}l_{2}^{2}\gamma_{1}\gamma_{2}}\;\mathrm{and}\;b_{5}=\frac{2D_{1}D_{2}}{l_{1}^{2}l_{2}^{2}\gamma_{1}\gamma_{2}}$$

The inverse transformation of Equation (51) yields [38]

(53) $$Q(t)=n_{0}A\frac{2D_{1}D_{2}}{\gamma_{1}l_{2}l_{1}^{2}}\left[\frac{1}{\alpha^{2}+\beta^{2}}+\frac{1}{\beta \sqrt{\alpha^{2}+\beta^{2}}}e^{-\alpha t}\sin(\beta t-\phi )\right]h(t),$$

(54) $$\alpha =\frac{b_{4}}{2},\;\beta =\sqrt{b_{5}-{\left(\frac{b_{4}}{2}\right)}^{2}},\;\mathrm{and}\;\phi =arctg\left(\frac{\beta }{-\alpha }\right)$$

Here, the cumulative drug amount exhibits markedly different short-time behavior compared to case (3), reflecting the decisive influence of the distal boundary condition when the backing layer is diffusively thin.

**Figure 3:**
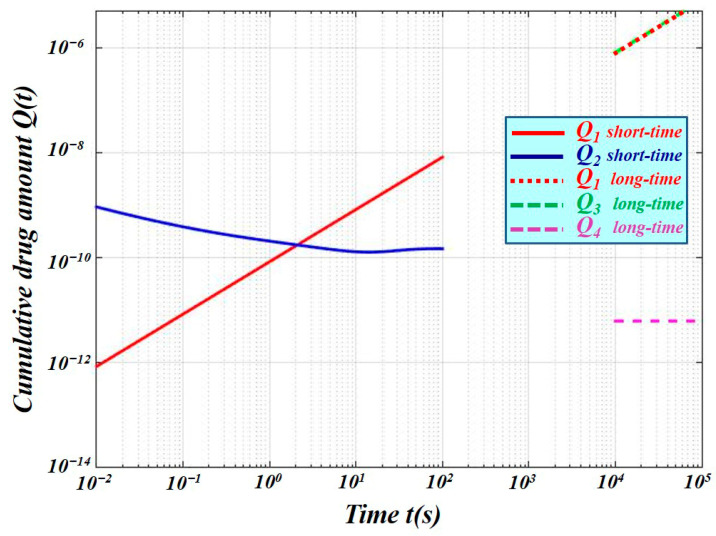
Cumulative drug amount *Q*(*t*) in short- and long-time regimes for different distal boundary conditions at the skin–systemic interface, computed using the diffusion-impedance model. Solid lines correspond to the short-time regime, while dashed lines correspond to the long-time regime. Red curves denote the reference case obtained by imposing a Dirichlet condition at the VE–dermis interface. In the short-time regime (diffusively thick backing layer), the distal boundary condition has negligible influence: uptake is primarily determined by the backing layer’s diffusivity and storage properties, while the skin–systemic interface remains effectively “invisible.” In contrast, in the long-time regime (diffusively thin backing layer), the distal boundary dominates the temporal evolution of *Q*(*t*), leading to marked differences between the ideal-sink assumption and the finite-layer predictions.

Using the parameters listed in Table 1 and assuming initial conditions *n*_0_ = 1 and *A* = 1, Figure 3 illustrates the time evolution of the cumulative drug amount *Q*(*t*) in the short- and long-time regimes for different distal boundary conditions, highlighting the regime-dependent behavior described in the previous section.

Solid lines in Figure 3 correspond to the short-time regime, while dashed lines correspond to the long-time regime. Red curves denote the reference case obtained by imposing a Dirichlet condition at the VE–dermis interface, and blue, green and violet lines correspond to derived diffusive impedance models.

When the backing layer is diffusively thick (short-time regime), the distal boundary condition does not influence the evolution of *Q*(*t*). A comparison of Equations (41) and (45) shows that, in this regime, the diffusivity and storage properties of the backing layer determine both the shape and magnitude of the cumulative drug amount, while the skin–systemic interface remains effectively “invisible” (Figure 3). The presence of an ideal sink (Dirichlet boundary condition) behind a thin barrier leads to a slower initial growth of *Q*(*t*).

As time progresses, the backing layer become diffusively thin (long-time regime). The situation then reverses: the backing layer no longer screens the influence of the distal boundary. As demonstrated by a comparison of Equations (51) and (53), the distal boundary condition now plays a dominant role in shaping the temporal evolution of *Q*(*t*) (Figure 3).

Let us now consider the effect of Robin boundary conditions. Robin conditions introduce an effective mass-transfer coefficient *h*, often interpreted as an experimental permeability parameter [9,10,13]. They account for finite layer conductance but reduce the backing layer to a single, static surface resistance. Taking *h* = *D*_2_/*l*_2_ and using Equations (13), (14), (35), (36) and (39) for the VE barrier, the cumulative uptake can be obtained via partial-fraction decomposition and inverse Laplace transform (Equations (55)–(57)):

(55) $$\bar{Q}(s)=An_{0}\frac{2D_{1}D_{2}}{l_{1}^{2}l_{2}\gamma_{1}}\frac{1}{s^{2}}\frac{1}{s+b_{6}}$$

where parameter *b*_6_ is defined by

(56) $$b_{6}=\frac{2\left(l_{1}D_{2}+l_{2}D_{1}\right)}{l_{2}l_{1}^{2}\gamma_{1}}$$

By applying partial-fraction decomposition, the inverse Laplace transform of Equation (55) yields Equation (57):

(57) $$Q(t)=An_{0}\frac{2D_{1}D_{2}}{l_{1}^{2}l_{2}\gamma_{1}}\frac{1}{b_{6}^{2}}\left(b_{6}t-1+e^{-b_{6}t}\right)h(t)$$

In the short-time limit, the slope of *Q*(*t*) depends on both layers’ diffusivities and thicknesses (via parameter *b*_6_), whereas in the long-time limit, the cumulative uptake lies between the predictions of the ideal-sink and full impedance models. This indicates that Robin conditions cannot reproduce the regime-dependent behavior captured by the diffusion-impedance model. By adjusting *h*, one can approximate either the short-time or long-time regime: tuning *h* to match long-time behavior potentially alters the early-time slope, making it closer to the ideal-sink case, while tuning for short-time uptake introduces discrepancies in the long-time limit.

**Figure 4:**
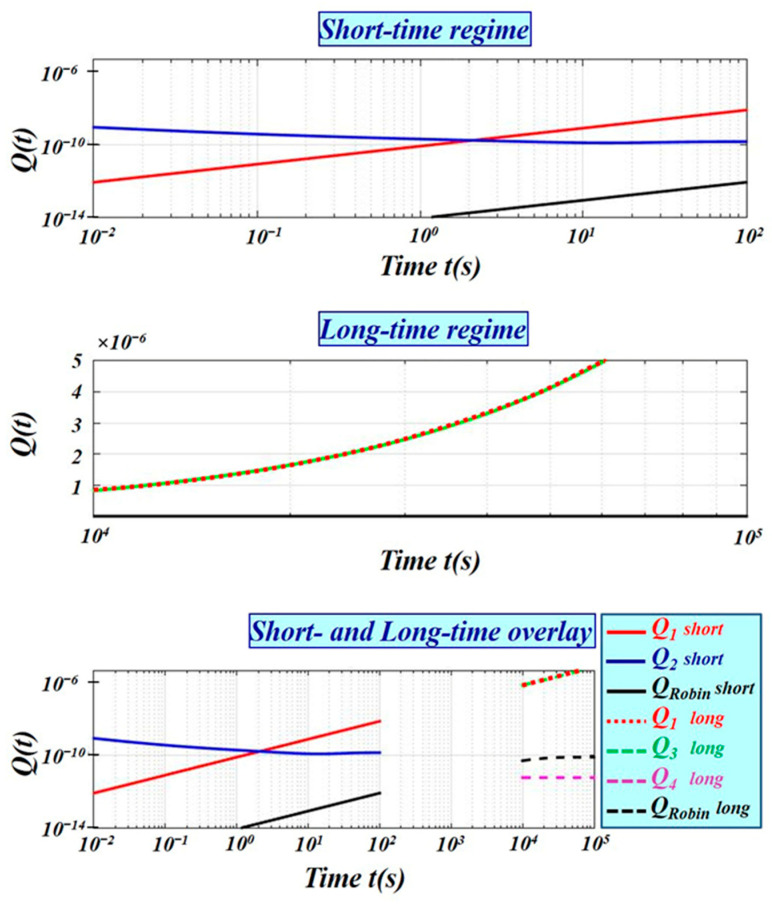
Comparison between the diffusion-impedance model and the Robin boundary approximation at the VE–dermis interface. Black curves represent the Robin boundary case (*h* = *D*_2_/*l*_2_); solid lines correspond to the short-time regime and dashed lines to the long-time regime. Red curves denote the Dirichlet (ideal-sink dermis) reference case. The remaining curves correspond to the diffusion-impedance model for different distal boundary conditions. The comparison demonstrates that a single Robin coefficient cannot simultaneously capture both temporal regimes. While Robin conditions account for finite layer properties, they neglect dynamic screening and the influence of distal boundaries, resulting in cumulative uptake values that are systematically smaller than those predicted by the impedance model. Specifically, when the distal boundary behaves as an ideal sink, the Robin approximation overestimates short-time uptake and underestimates long-time uptake, whereas for adiabatic distal boundaries, it underestimates short-time uptake and overestimates long-time uptake, highlighting the limitation of a single static mass-transfer coefficient in representing a finite backing layer.

Figure 4 compares the diffusion-impedance model and the Robin boundary approximation at the VE–dermis interface. Black curves represent the Robin case (*h* = *D*_2_/*l*_2_); solid lines correspond to short-time and dashed lines to long-time regimes. Red curves denote the Dirichlet reference case. The remaining curves correspond to the diffusion-impedance model for various distal boundary conditions.

## 4. Summary

### 4.1. Regime Dependence of Cumulative Drug Transport

The four regimes, summarized in Table 2, provide a clear overview of how backing-layer properties and distal boundary conditions govern cumulative drug uptake in classical approaches and in the suggested impedance-based approach.

These characteristic transport regimes not only clarify the physical role of the backing layer but also provide guidance for experimental design, such as selecting patch thickness, monitoring early- and long-time uptake, and interpreting ex vivo drug delivery data.

The temporal behavior of the cumulative drug amount *Q*(*t*) depends on whether the backing layer behaves as diffusively thick or thin relative to the observation time.

- Short-time regime (diffusively thick backing layer): The distal boundary condition does not influence *Q*(*t*). Uptake is controlled primarily by the backing layer’s diffusivity and storage capacity, while the distal boundary remains effectively invisible (Figure 3). The presence of an ideal sink (Dirichlet boundary) behind a thin barrier leads to slower initial uptake compared to a finite backing layer.
- Long-time regime (diffusively thin backing layer): The distal boundary becomes dominant, shaping the temporal evolution of *Q*(*t*) (Figure 3).
- Robin boundary conditions are often used to represent backing-layer effects through a single effective surface resistance. While they may approximate either short-time or long-time uptake, they cannot simultaneously capture both regimes. In the short-time limit, the backing layer screens the distal boundary, but Robin conditions treat it as a uniform resistance, failing to represent this dynamic screening (Figure 4). In the long-time limit, when the backing layer is thin, the distal boundary dominates, but Robin conditions still reduce the layer to a constant resistance, neglecting geometry-dependent impedance. Therefore, Robin formulations can at best provide partial agreement with the full diffusion-impedance model and may introduce significant errors if the distal boundary is adiabatic or non-ideal. By contrast, the impedance-based framework naturally captures both short- and long-time dynamics, linking cumulative uptake directly to backing-layer properties and distal boundary conditions.

### 4.2. The Sensitivity of Cumulative Uptake to Backing Layer Parameters

The sensitivity of cumulative uptake to backing layer parameters is governed primarily by the large diffusivity contrast between the epidermal barrier and the backing layer (*D*_2_ ≫ *D*_1_) and by the ratio of the very thin barrier of VE to the dermis layer (Table 1).

To quantify this coupling, one may introduce the dimensionless parameter

$$\Lambda =\frac{D_{1}D_{2}}{l_{1}l_{2}}$$

The dimensionless parameter Λ quantifies the relative transport rates and geometric thicknesses of the epidermal barrier and the backing layer. For Λ ≫ 1, the backing layer acts as a fast reservoir dynamically coupled to a slow barrier, effectively screening distal boundaries during the short-time regime, while still allowing their influence to emerge at long times, in contrast to the Dirichlet boundary condition.

### 4.3. Practical Implications for Transdermal Drug Delivery

This analysis highlights several practical insights:

1. Dynamic barrier properties: The effective permeability of the epidermal barrier is not intrinsic but emerges from the backing layer’s transport properties, its thickness, and distal boundary conditions.
2. Early-time uptake: Dominated by backing-layer diffusivity and storage; distal boundaries are largely invisible. Neglecting this can lead to over- or underestimation of drug delivery in experiments or simulations.
3. Long-time uptake: Sensitive to the distal boundary, which must be properly represented to predict steady-state behavior.

Scenarios where the impedance-based approach offers clear advantages:

- Ex vivo experiments with finite receiver volume, where ideal sink assumptions fail.
- Skin regions with slow dermal clearance (e.g., poorly vascularized areas).
- Controlled-release patches requiring accurate prediction of both early- and long-time dynamics.

These insights can guide both the design of controlled-release patches and the interpretation of uptake measurements in ex vivo or microfluidic experiments, ensuring that backing-layer effects are correctly captured.

## 5. Conclusions

In this work, we developed an analytical framework for transdermal drug delivery across multilayer skin membranes, modeling the system as a thin epidermal barrier dynamically coupled to a finite diffusive backing layer representing the dermis–hypodermis composite.

The key conceptual advance is treating the deeper layers as active transport elements rather than imposed boundary conditions. Their influence emerges naturally from intrinsic conductive, capacitive, and geometric properties. Diffusion impedance links interfacial concentration and flux directly, eliminating ad hoc interfacial assumptions.

Analytical solutions reveal a clear distinction between short- and long-time transport regimes, providing direct guidance for experimental measurements, data fitting, and the design of impedance-controlled systems:

- Short-time regime: Backing-layer transport dominates; distal boundaries are effectively invisible.
- Long-time regime: Distal boundaries dominate.
- Classical Dirichlet or Robin approaches fail to capture regime-dependent effects.

These classical boundary conditions arise only as limiting cases: Dirichlet corresponds to infinitely large backing-layer diffusivity, while Robin formulations represent a very thin backing layer between the barrier and an ideal sink at the distal boundary.

The framework is readily extensible to anomalous, fractional, or memory-dependent diffusion models, retaining the same analytical structure through the complex coefficient of propagation (Equation (13)) and characteristic impedance (Equation (14)) of each layer.

Future research will apply the framework to specific multilayer structures and experimental conditions, enabling quantitative benchmarking against real systems and refinement of model parameters, such as layer diffusivities, thicknesses, and mass-transfer coefficients, while building upon the practical implications discussed for early- and long-time transport regimes.

Overall, the impedance-based framework provides a unified, physically grounded, and analytically tractable approach to describe cumulative drug uptake, guide experimental interpretation, and support the design of multilayer transport systems. It establishes a direct link between multilayer transport dynamics and measurable uptake curves, enabling more accurate experimental interpretation and more reliable parameter identification.

## Figures and Tables

**Table 1:** Geometrical and diffusive properties of skin membrane layers [13,32,33,39].

	VE	Dermis + Hypodermis
Thickness [μm] [13,32,33]	50.8	987
Coefficient of diffusivity [m^2^s^−1^] [13,39]	3 × 10^−14^	3.61 × 10^−11^
Capacity [13]	0.14	1

**Table 2:** Summary of the four characteristic transport regimes, their controlling parameters, and sensitivity to distal boundary conditions.

Regime	Backing Layer	Distal Boundary	Dominant Mechanism	Sensitivity to BC
Short-time	Diffusively thick	Screened	Backing-layer storage & diffusivity	Low
Long-time	Diffusively thin	Visible	Distal boundary control	High
Dirichlet	Ideal sink	Invisible	Regime-indpendent	Not sensitive
Robin	Effective resistance	Ideal sink	Regime-independent	Via experimental parameter

## Data Availability

The raw data supporting the conclusions of this article will be made available by the authors on request.
